# Implementation of a hybrid lung health program for Northeast Texas: study protocol

**DOI:** 10.1186/s43058-026-00874-6

**Published:** 2026-02-19

**Authors:** Anastasia Rogova, Lorraine R. Reitzel, Lisa M. Lowenstein, Paul M. Cinciripini, Maggie Britton, Robert J. Volk, James G. Fox, Tzuan A. Chen, Jennifer A. Minnix

**Affiliations:** 1 Department of Health Services Research, The University of Texas MD Anderson Cancer Center, Houston, TX USA; 2 Department of Behavioral Science, The University of Texas MD Anderson Cancer Center, Houston, TX USA; 3 Department of Internal Medicine, University of Texas at Tyler Health Science Center, US-271, Tyler, TX 11937 USA; 4 Department of Psychological, Health, and Learning Sciences, University of Houston, McElhinney Hall, 3623 Cullen Blvd., Houston, TX USA; 5 HEALTH Research Institute, University of Houston, Martin Luther King Blvd., Houston, TX 4349 USA

**Keywords:** Lung cancer screening, Tobacco cessation, Shared decision-making, Community engagement, Cancer prevention

## Abstract

**Background:**

Lung cancer is the leading cause of cancer-related mortality in the United States. Lung cancer screening is recommended to individuals with a history of heavy cigarette smoking; it is proven to be effective in reducing lung cancer-related mortality, but the uptake remains low. In this project, we employ a hybrid model to ensure consistent screening for tobacco use and lung cancer screening eligibility, provision of tobacco cessation care, shared decision making for lung cancer screening, subsequent completion of screening, and navigation to follow-up care as needed, in Northeast Texas.

**Methods:**

The project is a partnership between a comprehensive cancer center, a university-affiliated hospital system, and a Federally Qualified Health Center. The intervention is designed as a hybrid lung cancer screening program, with a mix of centralized and decentralized features, to deliver high-quality tobacco cessation and lung cancer screening care for individuals in Northeast Texas. Major components of the intervention include training clinicians in shared decision making for lung cancer screening and tobacco cessation interventions; ensuring consistent screening for tobacco use and identification of patients eligible to lung cancer screening; provision of tobacco cessation interventions; and lung cancer screening completion support and navigation for further care, including follow-up testing and lung cancer treatment, as needed.

**Discussion:**

Through a hybrid care model, the project aims to enhance the quality of tobacco cessation care and shared decision making for lung cancer screening, increase screening uptake by eligible patients, and promote adherence to follow-up care and annual screening. The project also contributes to increased awareness of lung cancer and lung cancer screening in the community through dissemination and outreach strategies.

Contributions to the literature

- The protocol describes the implementation of a hybrid lung cancer screening program designed to systematically address both patient- and provider-level barriers and improve access to tobacco cessation care and lung cancer screening uptake.
- Findings of the project will contribute to better understanding of the role of hybrid models of lung cancer screening care delivery in rural and medically underserved areas affected by smoking-related illnesses.
- The project is guided by the Exploration, Preparation, Implementation and Sustainment framework and informs future efforts to raise awareness and promote sustainable delivery of lung cancer screening and tobacco cessation services in healthcare and community settings.

## Background

Lung cancer remains the leading cause of cancer-related mortality in the US and globally, with > 125,000 Americans dying from lung cancer annually [1]. Over 80% of lung cancer-related deaths are attributed to cigarette smoking, underscoring the importance of smoking cessation as a critical strategy to reduce the burden of lung cancer. Currently, ~ 11.5% of American adults smoke conventional cigarettes [2]. An effective strategy for reducing lung cancer mortality is screening persons with a history of heavy cigarette smoking using low-dose computed tomography (LDCT) imaging. Landmark trials, including the National Lung Screening Trial (NLST) [3] and the NELSON trial [4], have demonstrated that screening using LDCT imaging can significantly reduce lung cancer mortality through early detection. The US Preventive Services Task Force (USPSTF) currently recommends annual LDCT screening for individuals aged 50–80 years with a 20 pack-year smoking history who currently smoke or quit within the past 15 years [5]. The Centers for Medicare & Medicaid Services (CMS) covers lung cancer screening (LCS) with LDCT imaging, with a slight modification for eligibility (aged 50–77). Additionally, CMS mandates patient counseling about smoking cessation and shared decision making (SDM) using a patient decision aid before screening [6]. SDM counseling’s aim is to inform patients about risks and benefits of LCS and help them make a decision that aligns with their values and preferences. It also provides an opportunity to deliver evidence-based smoking cessation interventions to patients who smoke cigarettes, which are not otherwise consistently offered by providers at every healthcare encounter as recommended by clinical guidelines [7].

Although LCS is recommended by professional organizations and reimbursed by CMS and private insurance, only ~ 20% of eligible US patients are screened annually [5, 8]. Low screening rates have been attributed to barriers at multiple levels. Healthcare systems report the need for formal processes to identify patients eligible for screening, protocols for managing lung nodules and incidental findings, and navigation of patients to diagnostic testing and treatment when necessary. Providers report the need for greater clarity about coverage by CMS or private insurance, tools for patient education, training for clinic staff, and understanding about clinical guidelines [9]. A study of 125 healthcare clinics in Texas found that only 8.8% of them routinely refer patients for LCS; of the 23 Federally Qualified Health Centers (FQHCs) participating in the study, only 39.1% made regular LCS referrals [10]. Patients, meanwhile, often face barriers such as lack of awareness of LCS, difficulties accessing healthcare, fear of a cancer diagnosis, smoking-related and lung cancer stigma, and general mistrust of the healthcare system [11].

To optimize the delivery of LCS, different models have been implemented mostly following three approaches: centralized, decentralized, and hybrid. In a decentralized approach, primary care providers are responsible for completing nearly all functions of LCS, including SDM, smoking cessation counseling, ordering LDCT imaging, and coordinating follow-up care and adherence to annual screening. In centralized models, staff and clinicians trained in SDM and LCS guidelines coordinate most of these key functions, leading to a streamlined approach to managing LDCT findings, and the ability to navigate patients through the process of LCS. Hybrid models combine elements of both approaches, where for example, primary care providers can choose to complete some steps of LCS, such as initial conversations about screening including SDM, but they refer patients to a centralized program for LDCT imaging, managing nodules and other findings, follow-up care, and annual screening adherence [12–14].

Current guidelines do not recommend one delivery model over another and suggest that the choice depends upon local resources and other characteristics of each healthcare system and population they serve [13]. Recent data show some benefits of centralized models, particularly increased adherence to annual LCS [14–18]. Centralized programs have staff trained in SDM and LCS guidelines, streamlined approaches to managing LDCT findings, and resources to navigate patients through the process of LCS. The evidence is not clear, however, on what aspects of the centralized model contribute to better adherence and whether the same outcomes could be achieved in de-centralized models with additional community-based support, such as access to patient navigation [14, 19]. Furthermore, while a centralized approach leads to better annual adherence, evidence is lacking to suggest that it improves access to and initial uptake of LCS [14]. There are some concerns that wide adoption of a centralized delivery model could exacerbate health disparities by improving LCS only in larger, better resourced settings that provide care to individuals who are more likely to have better insurance and a higher socioeconomic status [14, 20]. More importantly, health systems may not have the resources to fully implement centralized programs, thus requiring alternative models to deliver high-quality LCS. Hybrid models can be effective in leveraging benefits of each approach, while being sensitive to local needs and contexts; however, such models may require more effort and resources to design and implement them in real-world clinical settings.

The routine provision of tobacco cessation services to patients in healthcare settings also faces challenges. A recent study of 86 healthcare centers in Texas indicated that while 70% of clinics routinely screened patients for tobacco use, even brief tobacco cessation interventions were inconsistently provided, being delivered in only 34% to 69% of cases [21]. Another study of 125 healthcare centers in Texas indicated that about 38% of them were unfamiliar with the Texas Tobacco Quitline [22], an empirically-supported source of tobacco cessation care [23]. Furthermore, despite clinical guidelines, a recent study that included 63 LCS facilities in Texas reported that most of these centers never referred patients who smoked to cessation services, provided cessation counseling, or recommended cessation medications [24]. Even though most adults who smoke cigarettes are interested in quitting [25], they often struggle to manage stress associated with quitting smoking, have limited social support, and experience inadequate access to smoking cessation care [26, 27]. Healthcare providers also report multiple barriers to providing cessation care, including lack of time, limited knowledge of interventions, competing priorities, and low organizational prioritization [26, 28].

Gaps in the implementation of evidence-based care for lung health are particularly pronounced in rural and medically underserved areas, where tobacco use rates remain high, quit rates are low, and access to LCS and tobacco cessation interventions are limited [29–32]. In the US, Texas has the largest rural population (~ 4.7 million) [33]. Data from 2018 indicated 28,000 smoking-related deaths in Texas and almost $9 billion in healthcare costs, emphasizing a significant effect on health and quality of life [34]. Moreover, the burden of tobacco use is not equally distributed across all areas of the state; Northeast Texas, the target area of this project, has significantly higher rates of cigarette use (18–20%) than the state does overall, as well as the country [2]. The region also leads the state in expected deaths from lung and bronchus cancers [35], making Northeast Texas a priority area for both the delivery of tobacco cessation interventions and the provision of LCS with subsequent navigation to cancer care [2, 34].

This project aims to address these critical care gaps by streamlining the delivery of evidence-based tobacco cessation and LCS care, including SDM counseling, for patients residing in Northeast Texas. We propose implementing a hybrid approach to promote consistent quality and adherence to the guidelines for LCS while relying on providers in participating clinics to identify eligible patients and conduct tobacco cessation and SDM counseling. Additionally, we incorporate telehealth options and access to the MD Anderson Quitline, which provides expanded smoking cessation support to individuals who qualify for LCS. By using this hybrid approach, we seek to leverage the benefits of a centralized model while remaining responsive to local needs with the ultimate objective of increasing Northeast Texans’ access to LCS.

## Methods

### Project goals and objectives

The overall goals of this project are to: (1) empower healthcare providers to screen patients for tobacco use and connect those interested in quitting with evidence-based cessation care; (2) support providers to engage in SDM counseling about LCS to all patients who meet eligibility criteria; (3) raise community awareness about LCS and LCS eligibility in Northeast Texas and actively disseminate the program to non-participating healthcare systems and clinics; and (4) navigate patients with abnormal LCS results to additional diagnostic testing and/or treatment, as needed. This work is guided by the Exploration, Preparation, Implementation and Sustainment (EPIS) framework [36], which provides a structured, systematic approach to identifying and addressing contextual barriers to evidence-based care at multiple levels, including those related to the outer context and inner contexts of the participating healthcare centers. This framework has been effectively applied to guide and evaluate implementation efforts within community-based healthcare settings, enhancing the understanding of processes and outcomes associated with implementation activities that involve collaborations between diverse stakeholders [36–38]. The program is additionally informed by Social Cognitive Theory (SCT), which emphasizes the reciprocal relationship between personal, behavioral, and environmental factors in shaping health-related behaviors [39]. SCT provides a framework for understanding and addressing key determinants of tobacco cessation and LCS participation, such as self-efficacy, outcome expectations, and social support that empower individuals to take an active role in their lung health, and their providers to actively participate in their care provision. Prior studies show that tobacco cessation education programs can contribute to increased tobacco-related knowledge among healthcare providers [40–42] and more consistent provision of tobacco cessation care overall [43–48].

**Fig. 1:**
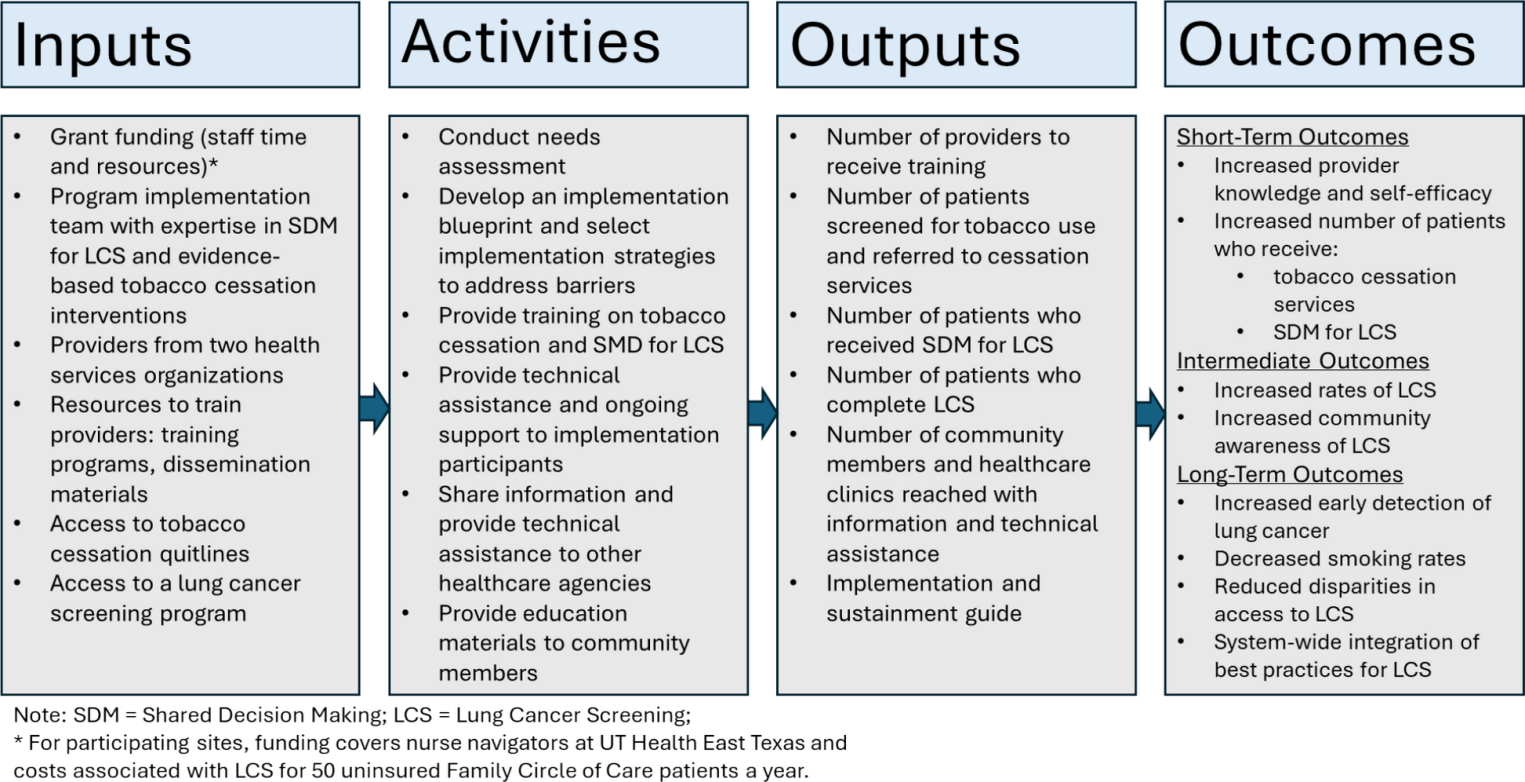
Logic model for the Lung Health Program for Northeast Texas illustrates the relationship between the program’s key inputs, activities, outputs, and expected outcomes

By applying these frameworks and leveraging the resources and capacities of a comprehensive cancer center, a university-affiliated hospital system serving rural Northeast Texas, and an FQHC, this multi-level, systemic approach aims to overcome known barriers, enhancing the quality of preventive healthcare delivery and improved lung health outcomes for high-risk populations. Figure 1 depicts the logic model outlining key inputs, activities, outputs, and expected outcomes of the program implementation.

The reporting of this protocol follows Standards for Reporting Implementation Studies (StaRI) [49] and the Template for Intervention Description and Replication (TIDieR) [50], to ensure transparency and facilitate implementation of effective interventions in real-world settings.

### Organizational and healthcare provider participants

This project involves researchers from The University of Texas MD Anderson Cancer Center (MDACC) and the University of Houston (UH). MDACC, an NCI-designated comprehensive cancer center, is the recipient of funding for this work and houses the MD Anderson Quitline and the Decision Science Core that will be leveraged for the project. The subcontract to UH supports the evaluation expert for this project.

The participating health services organizations are the UT Health East Texas (UT Health ET) and Family Circle of Care (FCC). UT Health ET is the largest healthcare provider in Northeast Texas with over 300 providers in more than 30 specialties who provide care across 50 clinics. Providers in the Departments of Family Medicine, Internal Medicine, and Pulmonary Medicine at UT Health ET will participate in program implementation. UT Health ET houses a Lung Nodule Program, which provides comprehensive LCS, evaluation, and guideline-based treatment of lung nodules. The program uses nurse navigators to facilitate LCS completion, LDCT imaging results reporting, as well as navigating patients with abnormal results to diagnostic testing and surveillance, and to treatment for patients with a lung cancer diagnosis. UT Health ET’s work on this project is supported via a subcontract, which includes funding to support our collaborators from the pulmonary clinic of UT Health ET, a MD who specializes in the treatment of lung disease and lung cancer screening and his lead nurse navigator. The subcontract also includes support for a clinical research coordinator and a nurse navigator for this project. FCC is an FQHC that includes six community-based clinics serving Tyler (4 clinics), Athens (1 clinic), and Jacksonville (1 clinic), Texas. FCC’s participating units include Family Medicine (embedded in 5 clinics), Obstetrics & Gynecology (4 clinics), and Internal Medicine (1 clinic). FCC is also a residency training site for the Department of Internal Medicine at UT Health ET.

### Hybrid lung health program core intervention components

The current protocol will use a hybrid delivery model to complement the extant Lung Nodule Program at UT Health ET by leveraging existing strengths at MDACC in training providers for SDM for LCS and in the provision of evidence-based tobacco cessation interventions. This approach combines benefits of a centralized LCS model, represented by the UT Health ET Lung Nodule Program, and those of decentralized models, where some elements of LCS are delivered by primary care providers (see Table 1). Providing SDM at the level of primary care settings addresses one of the concerns about a centralized model of LCS delivery, where SDM is delivered by the LCS program staff immediately before the LDCT appointment. Existing evidence suggests that SDM counseling is more meaningful and beneficial if it is provided before people reach LCS centers, improving access and ensuring a better uptake of screening, which is particularly important in settings serving rural populations [51]. The following aspects of the program will be delivered partly or completely virtually and are denoted* in Table 1: training in SDM and tobacco cessation interventions for providers; provision of SDM (may include virtual aids); provision of smoking cessation interventions for LCS eligible patients who smoke; community outreach, and continuing education. Core intervention components follow.

**Table 1:** Structure for the Lung Health Program for Northeast Texas

Program Component	UT Health East Texas & Family Circle of Care	MD Anderson Cancer Center
Identification of eligible patients for LCS	Implemented by participating providers though EHR	Provides tools for eligibility assessment (e.g. pack-year history calculator)
Training in SDM for providers*	Participating providers receive the training	MD Anderson Decision Support Lab provides in-person and on-demand SDM training
Provision of SDM about LCS for eligible patients*	Conducted by participating providers	MD Anderson Decision Support Lab provides patient decision aids; multiple formats and languages and low literacy materials
LCS results reporting; management of lung nodules and incidental findings; adherence to follow-up testing; adherence to annual screening	Conducted by UT Health East Texas Lung Nodule Program for all patients who complete LCS	n/a
Training in tobacco cessation interventions for clinicians*	Participating providers receive the training	MD Anderson project team develops and delivers the training
Identification of patients who use tobacco	Implemented by participating providers through EHR	n/a
Provision of patient education about smoking cessation and relapse prevention	Order materials based on patient demographics, and distribute to clinics for use	MD Anderson project team designs, orders, and ships patient education materials
Provision of tobacco cessation interventions – LCS eligible patients	Referral to the MD Anderson Quitline	MD Anderson Quitline provides smoking cessation care, including behavioral and pharmacological interventions
Provision of tobacco cessation interventions – non-LCS eligible patients	Referral to the Texas Tobacco Quitline	n/a
Community outreach to raise awareness of lung cancer and LCS*	UT Health East Texas team participates by attending community events, etc	MD Anderson’s project team attends community events, creates and distributes newsletters
Outreach with implementation and sustainment guide to non-participating healthcare centers and clinics	n/a	MD Anderson’s project team conducts outreach through emails, in-person visits, etc
Technical assistance for replication in non-participating programs*	n/a	MD Anderson’s project team provides technical assistance requested to support full or partial adoption for the program
Continuing education in tobacco cessation interventions for sustainment*	n/a	2 × monthly email sent to clinicians by MD Anderson’s study team, including continuing medical education resources

*EHR* Electronic Health Record, *SDM* Shared Decision Making, *LCS* Lung Cancer Screening

^*^The following aspects of the program will be delivered partly or completely virtually

#### Provider training on tobacco screening and intervention

All providers from the departments and clinics partnering on this project will receive training on tobacco use screening and cessation interventions. The training components of this program are informed by core constructs of SCT and designed to enhance self-efficacy and behavioral capability and build confidence in implementing the intervention. Training will be delivered on-site or virtually, synchronous or asynchronous as requested by stakeholders, with multiple training options of varied lengths provided, as needed, for maximal provider participation. We expect the asynchronous course, *Tobacco Dependence: Education and Training*, offering continuing medical education credits, will be widely used by providers due to its convenience [52]. The course was developed by the project team, leveraging their prior extensive experience in delivering tobacco dependence and cessation interventions, and has been widely available for use since March 2025. Initial results from test-takers to date (*N* = 101) show strong evidence that the training can effectively enhance provider knowledge, with a 54.8% knowledge gain overall as assessed by pre- and post-training assessments (unpublished data, available from authors). The training can, however, be modified to address the specific needs of locations participating in program implementation. Additionally, providers will be offered various tools to assist them to make tobacco use screening, brief intervention, and referral for cessation care part of their regular workflow. Providers will have opportunity to continue their training and education through a variety of options, many providing continuing education credits, that will be communicated to them on a regular basis.

#### Tobacco use interventions

All patients (aged 13 and up) will be screened for tobacco use; those who use tobacco will be provided with evidence-based tobacco cessation care. Tobacco use screening is currently a mandatory part of the patient’s electronic health record (EHR) at UT Health ET and FCC. Patients are screened for current and past tobacco use at every visit.

Patients who currently use tobacco and are interested in quitting will be referred to one of two quitlines for evidence-based tobacco use services inclusive of brief counseling and medications (nicotine replacement therapies, if not contra-indicated). Patients who meet eligibility for LCS will be referred to the MD Anderson Quitline, and patients who do not meet eligibility, including non-cigarette tobacco users, will be referred to the Texas Tobacco Quitline [53]. The different referral pathways for tobacco use treatment are reflective of the primary focus of the program on LCS-eligible patients. Providers from the departments and clinics enrolled in this project will be trained in referral procedures. All referrals for quitline care, including from FCC, will flow through the UT Health ET Lung Nodule Program, where connection with the appropriate quitline will be initiated based on LCS eligibility.

Connecting LCS-eligible patients to the MD Anderson Quitline enables comprehensive care with a dedicated Tobacco Treatment Specialist who follows the patient throughout their treatment (e.g., inclusive of dual nicotine replacement therapy provision, counseling, and a tailored motivational texting program). The MD Anderson Quitline, which has been funded by and leveraged in several research and quality improvement projects over the last decade [54], gathers and records data on patients’ quit attempts and treatment outcomes, inclusive of cotinine-verified abstinence, which is of interest for program evaluation and not part of the Texas Tobacco Quitline procedures. Non-LCS-eligible patients will receive dual nicotine replacement therapy and counseling by a trained specialist from the Texas Tobacco Quitline.

#### Lung cancer screening

**Fig. 2:**
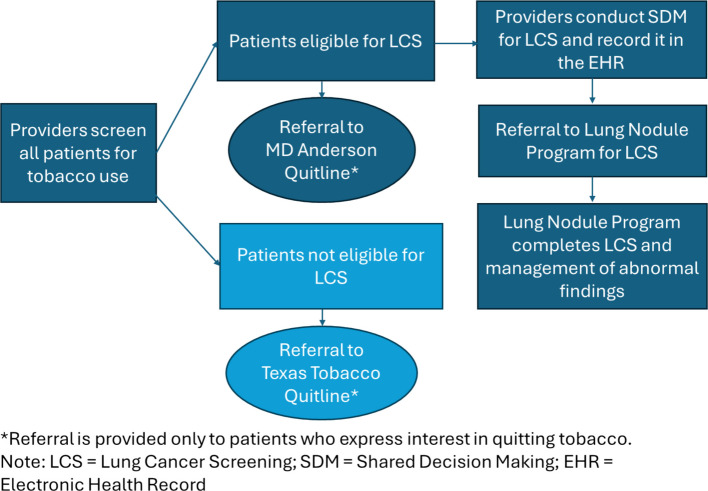
This figure illustrates the workflow of the program, outlining key processes for providers and patients in the Lung Health Program for Northeast Texas. Arrows indicate the sequence of the activities, and different colors represent different pathways for patients eligible and not eligible for lung cancer screening

Health service organizations participating in the project will evaluate all their patients with current or past history of cigarette smoking for LCS eligibility according to CMS criteria through the EHR [55]. For those eligible for LCS, their provider will initiate a SDM discussion with the patient that will be documented prior to referral. Following SDM counseling, patients who are interested in LCS will be referred to the UT Health ET Lung Nodule Program for screening and navigation to any follow-up procedures indicated (e.g., diagnostic testing, cancer treatment). Services for UT Health ET patients will be billed to the patients’ insurance; additionally, this project will provide funding for LCS for 50 uninsured FCC patients annually (150 total) through the UT Health ET Lung Nodule Program, where patients will receive support for diagnostic testing, surveillance, and treatment when indicated. Figure 2 illustrates the pathways of provider and patient engagement in the program.

#### LCS and SDM training

Providers will receive training on LCS and SDM in a manner that works best for their respective units, which will be determined within the exploration phase of this project. Various materials and resources were previously developed by the project team and Decision Science Core to train care providers, who showed large gains in core knowledge of lung cancer and eligibility for screening [56]. These resources will be leveraged for use in the current program, including various decision support tools and materials to facilitate delivery of high-quality SDM for LCS, an online eligibility calculator for determining pack-year smoking history [57], a video and printed patient decision aid, and discussion guides about LCS for use during provider encounters.

#### Community engagement

Broader community dissemination is an important goal of this work. To raise awareness of LCS and LCS eligibility in Northeast Texas communities, we will reach people through community engagement strategies, including through health fair engagements, community town halls, and health ministry collaborations, where we will engage and educate adults, providing them with lay-friendly and diversity-tailored health education materials on tobacco use cessation resources and LCS information. Various local, non-profit community organizations will be contacted with information about the program, LCS eligibility, and with health education materials that can be used to educate their clientele. We will leverage health education materials previously developed by the project team for this purpose.

#### Technical assistance

A step-by-step implementation and sustainment guide describing the enacted hybrid approach to LCS will be disseminated via email to leadership at healthcare systems and clinics across Texas. We will offer hands-on technical assistance to interested parties who wish to implement the whole or constituent parts of the program based on their needs or capacity.

## Implementation strategies and approach

Implementation strategies were selected following the Expert Recommendations for Implementing Change (ERIC) taxonomy guide [58] to support the implementation process and ensure it is tailored to the context and target recipients [59, 60]. The operationalization of the strategies is guided by the EPIS framework, which outlines four main phases of the implementation process: *exploration*, which involves assessing the needs, understanding the setting dynamics, workflow, facilitators, and barriers; *preparation*, focused on building capacity, developing resources, and setting expectations; *implementation*, which involves delivering the intervention, while monitoring fidelity and addressing any unanticipated challenges by using audit and feedback procedures to share providers’ progress; and *sustainment*, aimed at maximizing impact and continued use of intervention after the active implementation work is completed via iterative stakeholder consultations focused on quality assurance [36, 58, 61]. Table 2 outlines the key implementation strategies and their alignment with each phase of the EPIS framework.

**Table 2:** Implementation process components and objectives for the Lung Health Program for Northeast Texas

Phases of Implementation (EPIS)	Implementation Strategies and Components	Objectives
Exploration	Conduct local needs assessment; Complete pre-implementation surveys to assess for readiness and identify barriers and facilitators	Establish baseline knowledge, needs, barriers, and demographics for health service organization and participating clinics
Preparation	Sign Memorandum of Understanding between implementation participants	Achieve mutual understanding of process and expectations
	Organize internal implementation teams	Establish internal teams to support implementation and coordination between participants
	Develop a formal implementation blueprint with specific timeline	Discuss and set concrete steps for implementation; engage stakeholders in planning the program
	Provide technical assistance	Align internal systems with program expectations (i.e. EHR)
	Training programs preparation	Adapt training programs and dissemination materials to ensure responsiveness to local needs
	Develop education and dissemination materials	Provide access to resources to support implementation participants to deliver the program
Implementation	Conduct ongoing training in evidence-based tobacco cessation and SDM for LCS	Increase knowledge and skills required to implement the program, enhance self-efficacy of implementation participants
	Audit and provide feedback	Identify areas for further improvement and highlight successes; address barriers as needed
	Provide technical assistance	Provide support and consultations to healthcare partners to resolve issues that could delay the implementation
	Provide feedback	Support ongoing efforts to facilitate sustainability of the program and quality improvement opportunities
Sustainment	Provide technical assistance and training to community organizations	Maintain engagement and improve self-efficacy
	Provide continuous training opportunities	Support continuous learning and engagement to maintain knowledge and self-efficacy overtime
	Provide continuous training opportunities	Support continuous learning and engagement to maintain knowledge and self-efficacy overtime

During the *exploration* phase, we will assess participating organizations for readiness, identify barriers and facilitators, and conduct local needs assessment. These implementation strategies are selected to provide a better understanding of the inner and outer settings’ dynamics and workflow and evaluate the degree of readiness to implement the program. This formative process will also provide practical information needed to implement the program, such as the alignment of stakeholders’ EHR systems with data collection needs, including the documentation of smoking cessation care and SDM for LCS.

In the *preparation* phase, the research team will prepare healthcare leadership for active implementation by sharing an explicit summative document of program components, timeline, and expectations and assisting them in establishing internal implementation teams to ensure an effective execution of the project. A formal implementation blueprint will be tailored to participants’ needs and finalized during this phase.

Main strategies employed in the *implementation* phase will include ongoing training, technical assistance and consultation, and audit and feedback. Providers will receive training to screen for tobacco use, provide brief evidence-based motivational interviewing, cessation care, and relapse prevention interventions and quitline referrals, assess eligibility and facilitate SDM for LCS screening. Project partners will receive educational materials about smoking cessation and relapse prevention benefits for patients and clinical tools for providers. Audit and feedback procedures will include reviewing project documentation and patient data monthly and sharing findings with the leadership and providers to identify areas for further improvement and highlight successful elements of ongoing implementation on a quarterly basis. The project team will provide ongoing technical assistance and consultation to healthcare partners. Booster trainings to support staff and ensure their self-efficacy with care provision and referral processes will be provided as needed.

Data-driven adaptations to program implementation and materials will be used to maximize impact and identify mechanisms for *sustainment* of the intervention. Regular audits will ensure maintenance of procedures and inform feedback to leadership with recommendations for continued sustainment. Facilitation of learning opportunities, identification of local champions, and alignment of LCS and tobacco cessation care with existing quality improvement initiatives will support a continued uptake and sustainment of the program across participating sites.

### Dissemination

Dissemination strategies, aimed to achieve broader community engagement and increase awareness of tobacco intervention resources and LCS, are informed by Brownson et al. [62] and the utilization-focused surveillance framework for knowledge diffusion [63]. The dissemination plan comprises both passive and active strategies and entails the use of best practices for dissemination, including consideration of context, message, source, audience, and channel [62, 64, 65]. To reach *academic audience*, we will use open access publications and presentations. *Clinical and professional stakeholders* will be reached through targeted presentations, including webinars. To disseminate the program to *healthcare agencies*, we will use a step-by-step implementation and sustainment guide that includes a discussion of barriers to dissemination; a description of personnel and necessary resources to overcome barriers to program implementation; a description of expected outcomes; evaluation strategies with a sample evaluation plan; brief user-friendly case studies and narratives from our implementation experiences; FAQs; provider materials for point-of-decision use prompting; and quality management for sustainment. *Patient-facing* health promotion materials and patient decision aids relevant to tobacco use treatment and LCS will be developed and made available to community members. *Community* engagement strategies will include attendance of health fairs, virtual or onsite meetings with community groups, health ministry collaborations, regular newsletter distribution, and so on to provide lay-friendly and diversity-tailored health education materials for these audiences.

### Project goals and evaluation objectives

The current project has four overarching goals, each with several objectives. Table 3 describes program implementation goals and associated evaluation objectives, along with the types of data collected and their sources.

- Goal 1: Provide evidence-based tobacco use screening and cessation care for adult patients who smoke cigarettes. The objectives associated with Goal 1 are to ensure that 1) all participating providers receive at least one continuing medical education training to address tobacco use; 2) all adult patients who meet eligibility for LCS, currently use tobacco, and are interested in quitting are referred for tobacco cessation services through the MD Anderson Quitline and 15% of engaged patients achieve abstinence; and 3) all adult patients who currently use tobacco and are interested in quitting but do not meet eligibility for LCS are referred to the Texas Tobacco Quitline for tobacco cessation services.
- Goal 2: Provide SDM about LCS to all qualifying patients. Goal 2 objectives address LCS and include 1) continuing medical education about SDM for LCS to all participating providers; 2) SDM counseling for LCS to all eligible patients who are referred for LCS; and 3) 50% of patients referred for their first LCS complete it.
- Goal 3: Promote the program to community stakeholders and disseminate to healthcare systems and clinics. Objectives of Goal 3 involve 1) reaching community members with information about LCS eligibility and availability through passive and active dissemination of the program information and 2) contacting healthcare systems and clinics about the program with our implementation and sustainment guide and providing hands-on technical assistance and resources to support self-led implementation of program components in new healthcare systems and clinics.
- Goal 4: Navigate patients with abnormal screenings to diagnostic testing; those with cancer into treatment. Goal 4 includes two objectives that will ensure that 1) all patients whose screenings are abnormal will be navigated to and receive diagnostic testing and 2) all patients diagnosed with cancer and requiring cancer treatment will be navigated into treatment.

**Table 3:** Implementation of a Hybrid Lung Health Program for Northeast Texas: goals, data types and sources, and evaluation objectives

Goal	Data Collected	Data Source(s)	Evaluation Objective
1. Provide evidence-based tobacco use screening and dependence care for adult patients who smoke cigarettes	Number of providers who attend an education session	MD Anderson project team records	All primary care providers from the target departments and clinics participate in ≥ 1 training (virtual or in-person)
	Number of patients referred to cessation services	EHR records; MD Anderson project team records	100% of patients who currently smoke and are interested in quitting are referred to a quitline
	Number of LCS-eligible patients engaged in MD Anderson Quitline who are abstinent at end of treatment	MD Anderson Quitline team records	15% of engaged patients will achieve abstinence
2. Provide SDM about LCS to all qualifying patients	Number of providers who attend an education session	MD Anderson project team records	All providers from the target departments and clinics participate in ≥ 1 training (in-person or virtual)
	Number of patients with a documented SDM visit before first LCS	EHR records from UT Health East Texas and Family Circle of Care	100% patients who meet eligibility for LCS receive SDM for LCS
	Number of patients completed first LCS	EHR records from UT Health East Texas Lung Nodule Program	50% of referred patients complete first LCS
3. Promote the program to community stakeholders and disseminate to healthcare systems and clinics	Number of community members reached	MD Anderson project team records	Reach ≥ 180 community members through > 18 activities (e.g., event attendance, newsletters, etc.)
	Number of healthcare clinics/systems received technical assistance	MD Anderson project team records	Contact >/= 90 healthcare systems/clinics (e.g., emails, in-person visits, mail-outs)
			Provide technical assistance to support self-led implementation of >/= 1 program component/s in >/= 2 new healthcare systems/clinics
4. Navigate patients with abnormal screenings to diagnostic testing; those with cancer into treatment	Number of patients navigated to diagnostic testing after abnormal LCS results	EHR records from UT Health East Texas Lung Nodule Program	100% of patients with abnormal results navigated to diagnostic testing
	Number of patients diagnosed with lung cancer navigated to treatment	EHR records from UT Health East Texas Lung Nodule Program	100% of patients with cancer navigated into treatment

*EHR* Electronic Health Record, *LCS* Lung Cancer Screening, *SDM* Shared Decision Making

### Measurement and data collection

Data collection is structured to comprehensively assess both the implementation process and program outcomes. Incorporating constructs from SCT and key determinants from the EPIS framework, the approach aligns with the broader goal of the program evaluation across all objectives described above. Quantitative data, which will form the basis for evaluating project outcomes, will be obtained through participant surveys, electronic health records, and study-related documentation. Additionally, to further evaluate the implementation outcomes across all study goals, qualitative data will be collected through exit interviews with healthcare providers participating in the project. These interviews will focus on their experiences, perceptions, and engagement with the implementation of the project components.

UT Health ET and FCC will use their EHRs to assess the number of adult patients who are screened for tobacco use and referred to evidence-based tobacco dependence care (Goal 1); receive SDM for LCS; referred to LCS; complete their first LCS (Goal 2); navigated to diagnostic testing and treatment if diagnosed with cancer (Goal 4). Information about referrals and tobacco treatment engagement from the departments and clinics enrolled in this project will be collected by the Texas Tobacco Quitline and provided monthly to the implementation team as part of a larger, summative report of use. The MD Anderson Quitline reports will be used to collect information on patient enrollment, sessions completed, nicotine replacement therapy provided, and abstinence status at 3 and 6-months post-enrollment (Goal 1).

Study team records will be used to assess the number of providers who participate in education on evidence-based tobacco cessation interventions and education about SDM for LCS (Goals 1, 2). Using data from study-related records, we will also evaluate the number of community events in which we interactively provide information to community members and the number of community organizations to receive information about the program. We will also assess the number of healthcare systems and clinics to receive the step-by-step implementation and sustainment guide and hands-on technical assistance and resources to support self-led implementation of the program (Goal 3).

Pre-implementation surveys will be administered to the leadership of UT Health ET participating departments and FCC clinics to profile the providers and characterize the patient population. It will include measures such as the Organizational Readiness for Implementing Change (ORIC) measure [66] and the Lung Cancer Screening-12 (LCS-12) measure to evaluate participants’ knowledge and beliefs related to LCS [67]. To further evaluate the implementation outcomes for all aims, virtual post-implementation surveys will be administered to healthcare providers to assess acceptability of the program and solicit suggestions to improve efficiency and sustainability. Exit interviews with healthcare providers will be completed to assess the acceptability of the program and elicit feedback on the project’s impact on the clinical workflow and staff roles, as well as influential contextual factors (e.g., organizational culture).

### Data analysis

Quantitative outcomes will be assessed with descriptive statistics, frequencies, simple pre- to post-difference tests. Descriptive statistics will summarize clinic demographics, and the people and professionals reached and served. Non-parametric tests will be used to account for the small sample size of clinics whenever only clinic-level data are available. Wilcoxon signed rank sum tests and McNemar’s/Fisher’s exact tests will be used for clinic-level repeated continuous outcomes and categorical outcomes, respectively. The significance level will be set at *p*< 0.05. Data from all quantitative sources will be imported into a SAS file for data management. We will collect and analyze data beyond the objectives listed, including, for example, clinic moderators of change (as power may allow) to inform future work, with attention to collinearity, influential observations, robust standard errors adjusted for clustering within clinics (via mixed effect model), and adjustment for multiple comparisons, and effect size (Cohen’s d). Moderators of care delivery from our prior work include clinic size, clinic readiness to change, and provider smoking status [45, 68].

Qualitative data will be coded inductively with themes drawn from the data. Coding discrepancies will be discussed and reconciled within the team until consensus is reached, refining codes until a final coding frame is agreed upon and reapplied to all transcripts. Coding and analysis will proceed iteratively across each stage of data collection. Constant comparison of data will be used to refine themes, avoid redundancy, ensure fittingness of themes, and check accurate accounting of the dataset. Quantitative and qualitative data will be analyzed separately and merged at the end. Various types of integration will be used to merge the quantitative and qualitative data (e.g., creating a joint table).

## Discussion

In this protocol, we present a prevention-focused program aimed at increasing rates of tobacco cessation care provision and LCS completion by addressing both needs through a multifaceted, hybrid LCS delivery approach. The project aims to ensure consistent access to evidence-based tobacco screening and cessation care to all adult patients, emphasizing tobacco cessation as the main strategy of lung cancer prevention. For individuals meeting LCS eligibility, the program integrates SDM in participating clinics by training providers to facilitate informed decisions about LCS; this approach ensures better access and uptake of initial LCS and is more responsive to local needs and resources of these healthcare systems and their patients. For LCS completion, the program leverages the benefits of an existing Lung Nodule Program, which provides care and coordination for all aspects of LCS, ensuring consistent quality of care. Additionally, the project prioritizes community engagement to raise awareness about LCS across communities in Northeast Texas by actively disseminating the program to non-participating healthcare systems and clinics to broaden its reach.

The EPIS framework is used to guide the implementation and streamline the integration of SDM for LCS and tobacco cessation interventions in healthcare and community settings and ensure its sustainability by leveraging theory-informed and evidence-based implementation strategies and approaches. The aim of this applied approach is to use best practices of implementation science to ensure that evidence-based tobacco cessation and LCS interventions are integrated into routine clinical workflows of clinics participating in the project. Through these efforts, we seek to improve access to comprehensive lung cancer prevention and early detection services for populations in areas of Northeast Texas through creating a sustainable hybrid model of LCS delivery.

Potential limitations of this community-based LCS program include its generalizability and the feasibility of broader implementation. While we aim to tailor the program to the needs and capacities of participating partner organizations, it may not be directly transferable to other community settings with different resources, infrastructure, and patient populations. Though the current project includes significant implementation support with tailored training, technical assistance, and audit and feedback, it also incorporates a component of community dissemination, where other organizations are encouraged to adapt the program without ongoing support from the project team. These organizations that participate in a self-led implementation might experience additional barriers, such as a lack of training and dissemination materials tailored to their settings and patient populations, limited capacity, and competing demands, impacting the quality of program delivery and its outcomes. However, this project will seek to minimize these barriers by developing a more sustainable model of integrating LCS support into community healthcare systems, which will include an adaptable intervention and flexible implementation model leveraging available community resources.

## Data Availability

The datasets used and/or analyzed during the current study are available from the corresponding author on reasonable request.

## Abbreviations

CMS: Centers for Medicare & Medicaid Services
EHR: Electronic health record
EPIS: Exploration, Preparation, Implementation and Sustainment
ERIC: Expert Recommendations for Implementing Change
FCC: Family Circle of Care
FQHC: Federally Qualified Health Center
LDCT: Low-dose computed tomography
LCS: Lung cancer screening
MDACC: The University of Texas MD Anderson Cancer Center
SCT: Social Cognitive Theory
SDM: Shared decision making
UH: University of Houston
UT Health ET: UT Health East Texas

## References

[1] Siegel RL, Miller KD, Wagle NS, Jemal A. Cancer statistics, 2023. CA Cancer J Clin. 2023;73(1):17–48. 10.3322/caac.21763. 36633525 10.3322/caac.21763

[2] Cornelius ME, Loretan CG, Jamal A, et al. Tobacco product use among adults - United States, 2021. MMWR Morb Mortal Wkly Rep. 2023;72(18):475–83. 10.15585/mmwr.mm7218a1. 10.15585/mmwr.mm7218a1 PMC10168602 37141154

[3] Aberle DR, Adams AM, Berg CD, et al. Reduced lung-cancer mortality with low-dose computed tomographic screening - The National Lung Screening Trial Research Team. N Engl J Med. 2011;365(5):NEJMoa1102873. 10.1056/NEJMoa1102873 PMC4356534 21714641

[4] de Koning HJ, van der Aalst CM, de Jong PA, et al. Reduced lung-cancer mortality with volume CT screening in a randomized trial. N Engl J Med. 2020;382(6):503–13. 10.1056/NEJMoa1911793. 31995683 10.1056/NEJMoa1911793

[5] Moyer VA. Screening for lung cancer: U.S. preventive services task force recommendation statement. Ann Intern Med. 2014;160(5):330–8. 10.7326/M13-2771. 24378917 10.7326/M13-2771

[6] Centers for Medicare & Medicaid Services. Screening for lung cancer with Low Dose Computed Tomography (LDCT). 2022.

[7] Fiore MC, Jaen CR, Baker TB. Treating tobacco use and dependence: 2008 update. Clinical practice guideline. Rockville, MD, U.S. Department of Health and Human Services. Public Health Service. 2008.

[8] Henderson LM, Su IH, Rivera MP, et al. Prevalence of lung cancer screening in the US, 2022. JAMA Netw Open. 2024;7(3):e243190. 10.1001/jamanetworkopen.2024.3190. 38512257 10.1001/jamanetworkopen.2024.3190 PMC10958241

[9] Volk RJ, Foxhall LE. Readiness of primary care clinicians to implement lung cancer screening programs. Prev Med Rep. 2015;2:717–9. 10.1016/j.pmedr.2015.08.014. 26844142 10.1016/j.pmedr.2015.08.014 PMC4721428

[10] Britton M, Chen TA, Martinez Leal I, et al. Lung cancer screening eligibility and referral practices in Texas organizations serving people with substance use disorders. Cancers (Basel). 2023;15(7):2073. 10.3390/cancers15072073. 37046736 10.3390/cancers15072073 PMC10093429

[11] Hamann HA, VerHoeve ES, Carter-Harris L, Studts JL, Ostroff JS. Multilevel opportunities to address lung cancer stigma across the cancer control continuum. J Thorac Oncol: Off Publ Int Assoc Study Lung Cancer. 2018;13(8):1062–75. 10.1016/j.jtho.2018.05.014. 10.1016/j.jtho.2018.05.014 PMC6417494 29800746

[12] Alishahi Tabriz A, Neslund-Dudas C, Turner K, Rivera MP, Reuland DS, Elston LJ. How health-care organizations implement shared decision-making when it is required for reimbursement: The case of lung cancer screening. Chest. 2021;159(1):413–25. 10.1016/j.chest.2020.07.078. 32798520 10.1016/j.chest.2020.07.078 PMC7893305

[13] Mazzone PJ, Silvestri GA, Souter LH, et al. Screening for lung cancer: CHEST guideline and expert panel report. Chest. 2021;160(5):e427–94. 10.1016/j.chest.2021.06.063. 34270968 10.1016/j.chest.2021.06.063 PMC8727886

[14] Nunez ER, Triplette M. Addressing lung cancer screening disparities: what does it mean to be centralized? Ann Am Thorac Soc. 2022;19(9):1457–8. 10.1513/AnnalsATS.202206-495ED. 36048121 10.1513/AnnalsATS.202206-495ED PMC9447398

[15] Ezenwankwo E, Jones C, Nguyen DT, Eberth JM. Lung cancer screening adherence in centralized vs decentralized screening programs: a meta-analysis of us cohort studies among individuals with negative baseline results. Chest. 2025;168(3):797–809. 10.1016/j.chest.2025.04.033. 40345524 10.1016/j.chest.2025.04.033 PMC12489341

[16] Kim RY, Rendle KA, Mitra N, et al. Racial disparities in adherence to annual lung cancer screening and recommended follow-up care: a multicenter cohort study. Ann Am Thorac Soc. 2022;19(9):1561–9. 10.1513/AnnalsATS.202111-1253OC. 35167781 10.1513/AnnalsATS.202111-1253OC PMC9447384

[17] Sakoda LC, Rivera MP, Zhang J, et al. Patterns and factors associated with adherence to lung cancer screening in diverse practice settings. JAMA Netw Open. 2021;4(4):e218559. 10.1001/jamanetworkopen.2021.8559. 33929519 10.1001/jamanetworkopen.2021.8559 PMC8087957

[18] Smith HB, Ward R, Frazier C, Angotti J, Tanner NT. Guideline-recommended lung cancer screening adherence is superior with a centralized approach. Chest. 2022;161(3):818–25. 10.1016/j.chest.2021.09.002. 10.1016/j.chest.2021.09.002 34536385

[19] Spalluto LB, Lewis JA, LaBaze S, et al. Association of a lung screening program coordinator with adherence to annual CT lung screening at a large academic institution. J Am Coll Radiol. 2020;17(2):208–15. 10.1016/j.jacr.2019.08.010. 31499025 10.1016/j.jacr.2019.08.010 PMC7767624

[20] Simmons J, Gould MK, Woloshin S, Schwartz LM, Wiener RS. Attitudes about low-dose computed tomography screening for lung cancer: a survey of American Thoracic Society clinicians. Am J Respir Crit Care Med. 2015;191(4):483–6. 10.1164/rccm.201409-1747LE. 25679109 10.1164/rccm.201409-1747LE PMC4351598

[21] Jafry MZ, Martinez J, Chen TA, et al. Behavioral health care provider’s beliefs, confidence, and knowledge in treating cigarette smoking in relation to their use of the 5A’s intervention. Addictive behaviors reports. 2023:17. 10.1016/j.abrep.2023.100493 10.1016/j.abrep.2023.100493 PMC10279772 37347047

[22] Britton M, Rogova A, Chen TA, et al. Texas tobacco quitline knowledge, attitudes, and practices within healthcare agencies serving individuals with behavioral health needs: a multimethod study. Prev Med Rep. 2023;35:102256. 10.1016/j.pmedr.2023.102256. 37752980 10.1016/j.pmedr.2023.102256 PMC10518765

[23] Stead LF, Hartmann-Boyce J, Perera R, Lancaster T. Telephone counselling for smoking cessation. Cochrane Database Syst Rev. 2013(8):CD002850. 10.1002/14651858.CD002850.pub3 10.1002/14651858.CD002850.pub3 23934971

[24] Lowenstein LM, Nishi SPE, Lopez-Olivo MA, et al. Smoking cessation services and shared decision-making practices among lung cancer screening facilities: a cross-sectional study. Cancer. 2022;128(10):1967–75. 10.1002/cncr.34145. 35157302 10.1002/cncr.34145 PMC13137265

[25] VanFrank B. Adult smoking cessation — United States, 2022. MMWR Morb Mortal Wkly Rep. 2024;73:633. 10.15585/mmwr.mm7329a1 PMC11290909 39052529

[26] Presant CA, Ashing K, Raz D, et al. Overcoming barriers to tobacco cessation and lung cancer screening among racial and ethnic minority groups and underserved patients in academic centers and community network sites: the city of hope experience. J Clin Med. 2023;12(4):1275. 10.3390/jcm12041275. 36835811 10.3390/jcm12041275 PMC9965998

[27] Smith P, Quinn-Scoggins H, Murray RL, et al. Barriers and facilitators to engaging in smoking cessation support among lung screening participants. Nicotine Tob Res. 2024;26(7):870–7. 10.1093/ntr/ntad245. 38071660 10.1093/ntr/ntad245 PMC11190054

[28] Siddiqi AD, Britton M, Chen TA, et al. Tobacco screening practices and perceived barriers to offering tobacco cessation services among Texas health care centers providing behavioral health treatment. Int J Environ Res Public Health. 2022;19(15):9647. 10.3390/ijerph19159647. 35955001 10.3390/ijerph19159647 PMC9367734

[29] Doogan NJ, Roberts ME, Wewers ME, et al. A growing geographic disparity: rural and urban cigarette smoking trends in the United States. Prev Med. 2017;104:79–85. 10.1016/j.ypmed.2017.03.011. 28315761 10.1016/j.ypmed.2017.03.011 PMC5600673

[30] Roberts ME, Doogan NJ, Kurti AN, et al. Rural tobacco use across the United States: how rural and urban areas differ, broken down by census regions and divisions. Health Place. 2016;39:153–159. 10.1016/j.healthplace.2016.04.001 PMC4874850 27107746

[31] Parker MA, Weinberger AH, Eggers EM, Parker ES, Villanti AC. Trends in rural and urban cigarette smoking quit ratios in the US From 2010 to 2020. JAMA Netw Open. 2022;5(8):e2225326. 10.1016/j.healthplace.2016.04.001 10.1001/jamanetworkopen.2022.25326 PMC9350718 35921112

[32] Bittencourt L, Rubenstein D, Noonan D, McClernon FJ, Carroll DM. Smoking quit attempts and associated factors among rural adults who smoke daily in the United States. Nicotine Tob Res. 2024;26(7):948–53. 10.1093/ntr/ntad246. 38085266 10.1093/ntr/ntad246 PMC11190042

[33] United States Census Bureau Nation’s Urban and Rural Populations Shift Following 2020 Census. https://www.census.gov/newsroom/press-releases/2022/urban-rural-populations.html. Accessed 30 Mar 2025.

[34] Texas Department of State Health Services. 2023 Texas expected cancer cases and deaths. Prepared by Texas Cancer Registry, Cancer Epidemiology and Surveillance Branch, March 2023. 2023. https://www.dshs.texas.gov/texas-cancer-registry/cancer-statistics/expected-cancer-cases-deaths. Accessed 08–16–2023, 2023.

[35] Texas Department of State Health Services. 2023 Texas expected cancer cases and deaths. 2023. https://www.dshs.texas.gov/texas-cancer-registry/cancer-statistics/expected-cancer-cases-deaths. Accessed 21 Aug 2023.

[36] Moullin JC, Dickson KS, Stadnick NA, Rabin B, Aarons GA. Systematic review of the exploration, preparation, implementation, sustainment (EPIS) framework. Implement Sci. 2019;14(1):1. 10.1186/s13012-018-0842-6. 30611302 10.1186/s13012-018-0842-6 PMC6321673

[37] Aarons GA, Fettes DL, Sommerfeld DH, Palinkas LA. Mixed methods for implementation research: application to evidence-based practice implementation and staff turnover in community-based organizations providing child welfare services. Child Maltreat. 2012;17(1):67–79. 10.1177/1077559511426908. 22146861 10.1177/1077559511426908 PMC3841106

[38] Gadomski AM, Wissow LS, Palinkas L, Hoagwood KE, Daly JM, Kaye DL. Encouraging and sustaining integration of child mental health into primary care: interviews with primary care providers participating in Project TEACH (CAPES and CAP PC) in NY. Gen Hosp Psychiatry. 2014;36(6):555–62. 10.1016/j.genhosppsych.2014.05.013. 24973125 10.1016/j.genhosppsych.2014.05.013 PMC4240770

[39] Bandura A. Social foundations of thought and action: a social cognitive theory. Englewood Cliffs, N.J.: Prentice-Hall; 1986.

[40] Taing M, Nitturi V, Chen TA, et al. Implementation and outcomes of a comprehensive tobacco free workplace program in opioid treatment centers. Int J Environ Res Public Health. 2021;19(1):239. 10.3390/ijerph19010239. 35010499 10.3390/ijerph19010239 PMC8744608

[41] Correa-Fernández V, Wilson WT, Shedrick DA, et al. Implementation of a tobacco-free workplace program at a local mental health authority. Transl Behav Med. 2017;7(2):204–11. 10.3390/ijerph19010239. 28397160 10.1007/s13142-017-0476-2 PMC5526816

[42] Garey L, Neighbors C, Martinez Leal I, et al. Tobacco-related knowledge following a comprehensive tobacco-free workplace program within behavioral health facilities: identifying organizational moderators. Patient Educ Couns. 2019;102(9):1680–6. 10.1016/j.pec.2019.04.013. 31000352 10.1016/j.pec.2019.04.013 PMC6661000

[43] Siddiqi AD, Chen TA, Britton M, et al. Changes in substance use treatment providers’ delivery of the 5A’s for non-cigarette tobacco use in the context of a comprehensive tobacco-free workplace program implementation. Int J Environ Res Public Health. 2023;20(3):2730. 10.3390/ijerph20032730. 36768097 10.3390/ijerph20032730 PMC9914947

[44] Carter BJ, Siddiqi AD, Chen TA, et al. Educating substance use treatment center providers on tobacco use treatments is associated with increased provision of counseling and medication to patients who use tobacco. Int J Environ Res Public Health. 2023;20(5):4013. 10.3390/ijerph20054013. 36901024 10.3390/ijerph20054013 PMC10001967

[45] Le K, Chen TA, Martinez Leal I, et al. Organizational factors moderating changes in tobacco use dependence care delivery following a comprehensive tobacco-free workplace intervention in non-profit substance use treatment centers. Int J Environ Res Public Health. 2021;18(19):10485. 10.3390/ijerph181910485. 34639785 10.3390/ijerph181910485 PMC8507614

[46] Nitturi V, Chen T-A, Kyburz B, et al. Organizational characteristics and readiness for tobacco-free workplace program implementation moderates changes in clinician’s delivery of smoking interventions within behavioral health treatment clinics. Nicotine Tob Res. 2021;23(2):310–9. 10.1093/ntr/ntaa163. 32832980 10.1093/ntr/ntaa163 PMC7822101

[47] Correa-Fernández V, Wilson WT, Kyburz B, et al. Evaluation of the taking Texas tobacco free workplace program within behavioral health centers. Transl Behav Med. 2019;9(2):319–27. 10.1093/tbm/iby067. 10.1093/tbm/iby067 29955886

[48] Martinez Leal I, Chen T-A, Correa-Fernández V, et al. Adapting and evaluating implementation of a tobacco-free workplace program in behavioral health centers. Am J Health Behav. 2020;44(6):820–39. 10.5993/AJHB.44.6.7. 33081879 10.5993/AJHB.44.6.7

[49] Pinnock H, Barwick M, Carpenter CR, et al. Standards for Reporting Implementation Studies (StaRI) statement. BMJ. 2017;365:i6795. 10.1136/bmj.i6795. 10.1136/bmj.i6795 PMC5421438 28264797

[50] Hoffmann TC, Glasziou PP, Boutron I, et al. Better reporting of interventions: template for intervention description and replication (TIDier) checklist and guide. BMJ. 2014;348:g1687–g1687. 10.1136/bmj.g1687. 24609605 10.1136/bmj.g1687

[51] Volk RJ, Myers RE, Arenberg D, et al. The American Cancer Society National Lung Cancer Roundtable strategic plan: current challenges and future directions for shared decision making for lung cancer screening. Cancer. 2024;130(23):3996–4011. 10.1002/cncr.35382. 39302231 10.1002/cncr.35382 PMC12085948

[52] Tobacco dependence: education and training. https://mdanderson.cloud-cme.com/course/courseoverview?P=0&EID=40239. Accessed 20 Mar 2025.

[53] Texas Department of State Health Services. https://dshs.texas.gov/cessation-efforts/. Accessed 03 Nov 2025.

[54] Volk RJ, Lowenstein LM, Minnix JA, et al. 286 Using tobacco counselors to conduct shared decision making for lung cancer screening with patients who smoke: a cluster randomized trial. BMJ Evid Based Med. 2024;29(Suppl 1). 10.1136/bmjebm-2024-SDC.285

[55] Centers for Medicare & Medicaid Services. Screening for lung cancer with low dose computed tomography (LDCT) (CAG-00439R). 2022. https://www.cms.gov/medicare-coverage-database/view/ncacal-decision-memo.aspx?proposed=N&ncaid=304&fromTracking=Y&. Accessed 5 Dec 2024.

[56] Tan NQP, Volk RJ, Leal VB, et al. Tobacco quitline staffs’ knowledge and attitudes about connecting quitline callers to lung cancer screening educational materials. Cancer Med. 2024;13(13):e7443. 10.1002/cam4.7443. 38940442 10.1002/cam4.7443 PMC11212000

[57] Is lung cancer screening right for me? https://lungscreen.health/. Accessed 30 Mar 2025.

[58] Powell BJ, Waltz TJ, Chinman MJ, et al. A refined compilation of implementation strategies: results from the Expert Recommendations for Implementing Change (ERIC) project. Implement Sci. 2015;10(1):21. 10.1186/s13012-015-0209-1. 25889199 10.1186/s13012-015-0209-1 PMC4328074

[59] Fischer F, Lange K, Klose K, Greiner W, Kraemer A. Barriers and strategies in guideline implementation—a scoping review. Healthcare. 2016;4(3):36. 10.3390/healthcare4030036. 27417624 10.3390/healthcare4030036 PMC5041037

[60] Pereira VC, Silva SN, Carvalho VKS, Zanghelini F, Barreto JOM. Strategies for the implementation of clinical practice guidelines in public health: an overview of systematic reviews. Health Res Policy Syst. 2022;20(1):13. 10.1186/s12961-022-00815-4. 35073897 10.1186/s12961-022-00815-4 PMC8785489

[61] Aarons GA, Hurlburt M, Horwitz SM. Advancing a conceptual model of evidence-based practice implementation in public service sectors. Adm Policy Ment Health. 2011;38(1):4–23. 10.1007/s10488-010-0327-7. 21197565 10.1007/s10488-010-0327-7 PMC3025110

[62] Brownson RC, Eyler AA, Harris JK, Moore JB, Tabak RG. Getting the word out: new approaches for disseminating public health science. J Public Health Manag Pract. 2018;24(2):102–11. 10.1097/phh.0000000000000673. 28885319 10.1097/PHH.0000000000000673 PMC5794246

[63] Green LW, Ottoson JM, García C, Hiatt RA. Diffusion theory and knowledge dissemination, utilization, and integration in public health. Annu Rev Public Health. 2009;30(1):151–74. 10.1146/annurev.publhealth.031308.100049. 19705558 10.1146/annurev.publhealth.031308.100049

[64] Research protocol: communication and dissemination strategies to facilitate the use of health-related evidence. Effective Health Care Program, Agency for Healthcare Research and Quality, Rockville, MD. https://effectivehealthcare.ahrq.gov/products/medical-evidence-communication/research-protocol. Accessed 30 Mar 2025.

[65] Yamey G. Scaling up global health interventions: a proposed framework for success. PLoS Med. 2011;8(6):e1001049. 10.1371/journal.pmed.1001049. 21738450 10.1371/journal.pmed.1001049 PMC3125181

[66] Weiner BJ. A theory of organizational readiness for change. Implement Sci. 2009;4:67. 10.1186/1748-5908-4-67. 19840381 10.1186/1748-5908-4-67 PMC2770024

[67] Housten AJ, Lowenstein LM, Leal VB, Volk RJ. Responsiveness of a brief measure of lung cancer screening knowledge. J Cancer Educ. 2018;33(4):842–6. 10.1007/s13187-016-1153-8. 27966194 10.1007/s13187-016-1153-8 PMC5471134

[68] Siddiqi AD, Carter BJ, Chen TA, et al. Initial leadership concerns and availability of tobacco cessation services moderate changes in employee-reported concerns about tobacco-free workplace policy implementation over time. Transl Behav Med. 2024;14(7):394–401. 10.1093/tbm/ibae019. 38757794 10.1093/tbm/ibae019 PMC11208289

